# Neutrophil to lymphocyte ratio as a predictor of response to neoadjuvant chemotherapy and survival in oesophageal adenocarcinoma

**DOI:** 10.1002/bjs5.50277

**Published:** 2020-03-31

**Authors:** A. G. M. T. Powell, C. Chin, A. H. Coxon, A. Chalishazar, A. Christian, S. A. Roberts, W. G. Lewis

**Affiliations:** ^1^ Division of Cancer and Genetics Cardiff University Cardiff UK; ^2^ Department of Surgery University Hospital of Wales Cardiff UK; ^3^ Department of Pathology University Hospital of Wales Cardiff UK; ^4^ Department of Radiology University Hospital of Wales Cardiff UK

## Abstract

**Background:**

Inflammation has an important role in cancer survival, yet whether serum markers of inflammation predict response to potentially curative neoadjuvant chemotherapy (NAC) in oesophageal adenocarcinoma (OAC) is controversial. This study aimed to determine whether the systemic inflammatory response (SIR) is associated with response to NAC and survival.

**Methods:**

Consecutive patients with OAC planned for surgery with curative intent received blood neutrophil and lymphocyte measurements at diagnosis to calculate the neutrophil to lymphocyte ratio (NLR). Pathological variables including pTNM stage, differentiation, vascular invasion and Mandard tumour regression grade (TRG) were recorded. TRGs 1 and 2 were taken to represent a good response, and the primary outcome was overall survival.

**Results:**

During follow‐up of 136 patients, 36 patients (26·5 per cent) had recurrence and 69 (50·7 per cent) died. Receiver operating characteristic (ROC) curve analysis of NLR before NAC predicted poor TRG (area under the ROC curve 0·71, 95 per cent c.i. 0·58 to 0·83; *P* = 0·002). In univariable analysis, pT category (*P* < 0·001), pN category (*P* < 0·001), poor differentiation (*P* = 0·006), margin positivity (*P* = 0·001), poor TRG (*P* = 0·014) and NLR (dichotomized at 2·25; *P* = 0·017) were associated with poor overall survival, and NLR retained independent significance in multivariable analysis (hazard ratio 2·26, 95 per cent c.i. 1·03 to 4·93; *P* = 0·042).

**Conclusion:**

The pretreatment NLR was associated with a pathological response to NAC and overall survival in patients with OAC.

**Antecedentes:**

La inflamación juega un importante papel en la supervivencia por cáncer, aunque aún no se sabe si los marcadores séricos de inflamación predicen la respuesta a la quimioterapia neoadyuvante (*neoadjuvant chemotherapy*, NAC) potencialmente curativa en el adenocarcinoma de esófago (*oesophageal adenocarcinom*a, OAC). Este estudio se propuso determinar si la respuesta inflamatoria sistémica (*systemic inflammatory response*, SIR) estaba asociada con la respuesta a la NAC y a la supervivencia.

**Métodos:**

A pacientes consecutivos con OAC en los que se planificó cirugía con intención curativa se les determinó neutrófilos y linfocitos en sangre en el momento del diagnóstico para calcular la tasa neutrófilo‐linfocito (*neutrophil‐lymphocyte ratio*, NLR). Se registraron variables patológicas que incluían el estadio pTNM, diferenciación tumoral, invasión vascular y grado de regresión tumoral (*tumour regression grade*, TRG) de Mandard. Los grados TRG 1 y 2 fueron considerados como una buena respuesta y el resultado primario fue la supervivencia global (*overall survival*, OS).

**Resultados:**

Durante el seguimiento de 136 pacientes, 36 pacientes (26,5%) presentaron recidiva y 69 pacientes (50,7%) fallecieron. El análisis de las características operativas del receptor (*receiver‐operator‐characteristic*, ROC) de NLR antes de la NAC predijo una pobre TRG (área bajo la curva ROC, AUC 0,71, i.c. del 95% 0,58‐0,83, *P* = 0,002). En el análisis univariable, el estadio pT (*P* < 0,001), el estadio pN (*P* < 0,001), una pobre diferenciación tumoral (*P* = 0,006), un margen positivo (*P* = 0,001), una pobre TRG (*P* = 0,014) y la NLR (dicotomizada a 2,25, *P* = 0,017) se asociaron con una pobre OS, pero solamente la NLR (cociente de riesgos instantáneos, *hazard ratio*, HR 2,28, i.c. del 95% 1,03‐4,93, *P* = 0,042) conservó la significación estadística como variable independiente en el análisis multivariable.

**Conclusión:**

La NLR antes del tratamiento se asoció con respuesta patológica del OAC a la NAC y OS.

## Graphical abstract

The neutrophil to lymphocyte ratio, dichotomized at 2·25, was strongly associated with Mandard tumour regression grade in patients who had neoadjuvant chemotherapy for oesophageal adenocarcinoma. The systemic inflammatory response may offer a novel therapeutic target for patients at risk of poor response.

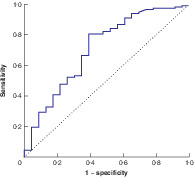

A simple tool to aid decision‐making

## Introduction

In the West, most patients who are offered attempted curative therapy for oesophageal adenocarcinoma (OAC) will undergo a multimodal treatment involving either neoadjuvant chemotherapy (NAC) followed by surgery^1^, perioperative chemotherapy^2^, or neoadjuvant chemoradiotherapy^3^. Despite evidence of survival benefit, a meta‐analysis^4^ comparing NAC with surgery alone in 2062 patients found only a 5·1 per cent absolute 2‐year survival advantage after NAC, because only a small minority experienced a significant pathological response. In a multicentre cohort study^5^, a clinically meaningful local response to NAC was restricted to the 14·8 per cent of patients with a tumour regression grade (TRG) of 1–2.

Inflammation is now widely recognized as a feature of many cancers^6^. Among a variety of inflammatory markers, derivative biomarkers – neutrophil to lymphocyte ratio (NLR), platelet to lymphocyte ratio (PLR), neutrophil to platelet score, and the modified Glasgow Prognostic Score (mGPS) – have been reported to be associated with poor survival^7^, ^8^, ^9^.

The aim of this study was to determine whether clinically readily available serum markers of inflammation obtained from routinely performed patient screening blood profiles might predict response to potentially curative NAC in OAC, and whether there was a subsequent relationship with survival after potentially curative oesophagectomy.

## Methods

To test the proposed hypotheses, a single cohort of patients diagnosed with OAC between 1 January 2010 and 31 August 2018 was recruited, and included patients with radiological TNM stage I–III deemed amenable to treatment with curative intent. All patients were managed by a multidisciplinary specialist team (MDT) with an interest in oesophageal cancer, including clinical nurse specialists, gastroenterologists, surgeons, oncologists, radiologists, anaesthetists and pathologists^10^. Management plans were individually tailored according to both patient and disease factors. Staging was done by means of CT, endoscopic ultrasonography, CT–PET and staging laparoscopy as appropriate. The South‐East Wales MDT treatment algorithms for oesophageal carcinoma have been described previously^11^, ^12^, ^13^.

The majority of patients received two cycles of cisplatin 80 mg/m^2^ and 5‐fluorouracil 1000 mg/m^2^ for 4 days. A minority received three cycles of epirubicin 50 mg/m^2^, cisplatin 60 mg/m^2^ and 5‐fluorouracil 200 mg/m^2^ or capecitabine 625 mg/m^2^ (ECF/X regimen). Definitive chemoradiotherapy was offered to patients with localized squamous cell carcinoma and those with adenocarcinoma deemed unsuitable for surgery because of disease extent and/or medical co‐morbidity^14^, ^15^.

The standard surgical approach was subtotal transthor‐acic oesophagectomy (TTO), as described by Lewis^16^ and Tanner^17^. Transhiatal oesophagectomy (THO), as described by Orringer^18^, was used selectively in patients with adenocarcinoma of the lower third of the oesophagus who had significant cardiorespiratory co‐morbidity, cT1–3 N0 disease. A modified extended D2 lymphadenectomy (preserving the pancreas and spleen where possible) was performed.

Ethical approval was sought, but the chair of the Cardiff and Vale University Health Board ethics committee confirmed that individual patient consent was not required to report clinical outcomes alone, and no formal approval was necessary.

### Clinicopathological characteristics

Tumours were staged using the seventh edition of the AJCC/UICC TNM staging system. Pathological factors were recorded from pathology reports issued at the time of surgery, and included tumour differentiation, margin status and the number of lymph nodes with and without metastasis. The TRG was quantified using the Mandard system^19^ by a histopathologist with a special interest in oesophagogastric cancer. Briefly, Mandard TRGs range from 1 to 5, based on the ratio of fibrosis to viable cancerous cells^5^, ^19^. In keeping with the Oesophageal Cancer Clinical And Molecular Stratification (OCCAMS) reporting methodology, TRGs of 1 and 2 were considered to constitute good response, with TRGs of 3, 4 and 5 constituting poor response^5^.

Routine laboratory measurements of haemoglobin, whole white cell count, neutrophil, lymphocyte and platelet counts at the time of diagnosis were recorded. Derivate measurements of systemic inflammation were constructed by calculating the NLR and PLR^7^, ^20^.

Patients were followed up at regular intervals of 3 months in the first year and 6 months thereafter. In the event that patients developed symptoms suggestive of recurrent disease, investigations were undertaken sooner. Follow‐up surveillance was conducted for 5 years or until death, whichever was sooner. Overall survival was calculated from time of diagnosis to the date of death or censoring. Disease‐free survival was measured from the date of surgery to the date of recurrence or censoring. The time of recurrence was taken as the date of the confirmatory investigation, on an intention‐to‐treat basis. Death certification was obtained from the Office for National Statistics via the Cancer Network Information System Cymru.

### Statistical analysis

Grouped data were expressed as median (i.q.r.) values, and non‐parametric methodology was used throughout. Receiver operating characteristic (ROC) curve analysis was employed to assess the predictive value of continuous variables with the primary outcome measure. ROC analysis was also used to determine dichotomization thresholds for poor Mandard TRGs, as described by Youden^21^. Univariable and multivariable logistical regression analysis was used to identify independent associations of categorical variables with poor Mandard TRGs. Variables with *P* < 0·100 were included in the model using backward conditional methodology. Patient demographics were analysed between the treatment modalities by means of χ^2^ or non‐parametric tests, including the Mann–Whitney *U* test. These tests were also employed in the analysis of disease recurrence and time to recurrence for the treatment groups.

Overall survival was measured from the date of diagnosis, and disease‐free survival from date of surgery. This approach was adopted in the randomized trials to allow for the variable interval to surgery after diagnosis, depending on whether NAC was prescribed^22^. As in the trials, events resulting in a failure to complete curative treatment, such as not proceeding to surgery, open and close laparotomy, palliative resection and in‐hospital mortality, were assumed to have occurred at this landmark time, in order to maintain the intention‐to‐treat analysis. Cumulative survival was calculated according to the Kaplan–Meier method, with differences between groups analysed using the log rank test. A univariable analysis examining factors influencing survival was performed initially by the life‐table method of Kaplan and Meier, and factors with associations found to be significant at the *P* < 0·010 level were retained in a Cox proportional hazards model using backward conditional methodology to assess the prognostic value of individual variables.

All statistical analysis was performed in SPSS® Statistics v25.0.0.0 (IBM, Armonk, New York, USA) with extension R.

## Results

A total of 136 patients with OAC were identified and underwent surgery after NAC; the operative approach was open in 120 patients with 16 patients undergoing laparoscopically assisted surgery. Details of patients' clinicopathological factors can be found in *Table* 1. Their median age was 68 (i.q.r. 63–73) years; 106 (77·9 per cent) were men and 30 (22·1 per cent) were women. Twenty‐three patients (16·9 per cent) had a good pathological response to NAC (TRG 1–2); the Mandard TRG groupings were: TRG 1, 18 (13·2 per cent); TRG 2, five (3·7 per cent); TRG 3, six (4·4 per cent); TRG 4, 56 (41·2 per cent); and TRG 5, 51 (37·5 per cent). During follow‐up, 36 patients (26·5 per cent) developed cancer recurrence and 69 (50·7 per cent) died. Median follow‐up of survivors was 27 (range 6–60) months. Around two‐thirds of the patients were followed up for at least 5 years or until death.

**Table 1:** Clinicopathological patient factors

	No. of patients (*n* = 136)
**Age (years)**	
< 65	46 (33·8)
66–75	68 (50·0)
> 75	22 (16·2)
**Sex ratio (F** : **M)**	30 : 106
**Operative approach**	*n* = 113
TTO	71 (62·8)
THO	42 (37·2)
**Neutrophil** : **lymphocyte ratio**	
< 2·25	36 (26·5)
≥ 2·25	100 (73·5)
**pT category**	
pT0	11 (8·1)
pT1	10 (7·4)
pT2	10 (7·4)
pT3	69 (50·7)
pT4	13 (9·6)
No resection	23 (16·9)
**pN category**	
pN0	35 (25·7)
pN1	34 (25·0)
pN2	26 (19·1)
pN3	18 (13·2)
No resection	23 (16·9)
**Mandard TRG**	
Good	23 (16·9)
Poor	113 (83·1)
**Differentiation**	
Well or moderate	64 (47·1)
Poor	72 (52·9)
**CRM**	
Negative	58 (42·6)
Positive	55 (40·4)
No resection	23 (16·9)
**Lymph node yield**	*n* = 113
< 15	43 (38·1)
≥ 15	70 (61·9)

Values in parentheses are percentages. TTO, transthoracic oesophagectomy; THO, transhiatal oesophagectomy; TRG, tumour regression grade; CRM, circumferential resection margin.

**Figure 1:**
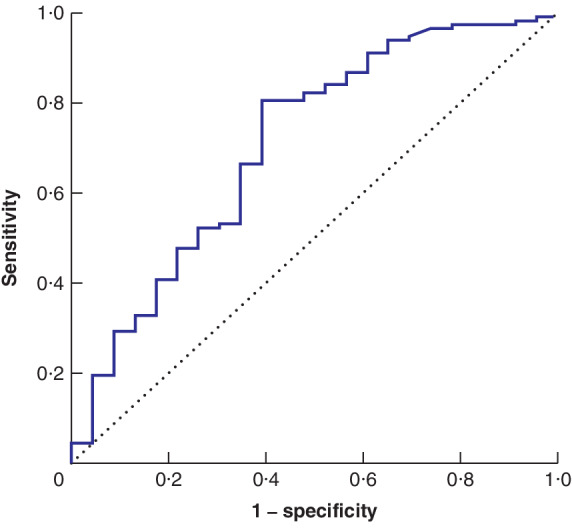
**Receiver operating characteristic (ROC) curve analysis of neutrophil to lymphocyte ratio and Mandard tumour response grade** Area under the ROC curve (AUC) = 0·71 (95 per cent c.i. 0·58 to 0·83; *P* = 0·002).

Baseline and area under the ROC curve (AUC) values for markers of the systemic inflammatory response (SIR) are shown in *Table* 2. The median value for NLR was 3·00 (i.q.r. 2·15–3·89). NLR was strongly associated with a poorer Mandard TRG (AUC 0·71, 95 per cent c.i. 0·58 to 0·83; *P* = 0·002) (*Fig*. 1). Using the Youden index, the optimal dichotomization threshold was 2·25, with 70·5 per cent considered to have a raised NLR. This gave sensitivity and specificity values of 80·5 and 60·9 per cent respectively. To adjust for potential confounding, a binary logistic regression model was developed to include the clinical factors available to the MDT at the point of commencing neoadjuvant therapy (*Table* 3).

**Table 2:** Association between pretreatment markers of systemic inflammatory response and poor Mandard tumour regression grade

		No. of patients with marker level†			ROC analysis	
	Concentration*	Low*	Normal	High	AUC	*P*
Haemoglobin (g/l)	138 (128–148)	33	103	0	0·47 (0·33, 0·62)	0·699
White cell count (× 10^9^/l)	7·6 (6·2–8·1)	2	124	10	0·59 (0·46, 0·72)	0·158
Neutrophil count (× 10^9^/l)	5·0 (3·9–6·1)	2	118	16	0·68 (0·56, 0·80)	0·008
Lymphocyte count (× 10^9^/l)	1·7 (1·3–2·1)	8	123	5	0·40 (0·25, 0·54)	0·115
Platelet count (× 10^9^/l)	281 (233–330)	3	123	10	0·52 (0·40, 0·65)	0·691
Neutrophil to lymphocyte ratio	3·00 (2·15–3·89)				0·71 (0·58, 0·83)	0·002
Platelet to lymphocyte ratio	160 (122–198)				0·66 (0·52, 0·79)	0·019

Values in parentheses are 95 per cent confidence intervals unless indicated otherwise;

* values are median (i.q.r.).

† Based on local thresholds.

AUC, area under the receiver operating characteristic (ROC) curve.

**Table 3:** Logistic regression analysis of preoperative factors associated with poor Mandard tumour regression grade

	Univariable analysis		Multivariable analysis	
	Odds ratio	*P*	Odds ratio	*P*
Age (< 65 *versus* 66–75 *versus* > 75 years)	1·11 (0·58, 2·15)	0·753		
Sex (F *versus* M)	1·31 (0·47, 3·68)	0·610		
Differentiation (well/moderate *versus* poor)	17·09 (3·82, 76·55)	< 0·001	15·92 (3·42, 74·02)	< 0·001
cTNM (1 *versus* 2 *versus* 3 *versus* 4)	1·38 (0·76, 2·48)	0·289		
Neutrophil to lymphocyte ratio (< 2·25 *versus* ≥ 2·25)	6·43 (2·47, 16·77)	< 0·001	5·86 (2·03, 16·92)	0·001

Values in parentheses are 95 per cent confidence intervals.

The relationship between clinicopathological factors and overall survival is shown in *Table* 4, and that between clinicopathological factors and disease‐free survival in *Table* 5. Five‐year overall survival rates for patients with a low and high NLR were 50 and 20 per cent, respectively. Five‐year disease‐free survival rates for low and high NLR were 80 and 40 per cent, respectively.

**Table 4:** Cox proportional hazards analysis of factors associated with overall survival

	Univariable analysis		Multivariable analysis	
	Hazard ratio	*P*	Hazard ratio	*P*
Age (< 65 *versus* 66–75 *versus* > 75 years)	0·87 (0·58, 1·30)	0·494		
Sex (F *versus* M)	1·13 (0·57, 2·21)	0·731		
Operative approach (TTO *versus* THO)	1·25 (0·69, 2·24)	0·462		
Neutrophil to lymphocyte ratio (< 2·25 *versus* ≥ 2·25)	2·33 (1·16, 4·68)	0·017	2·26 (1·03, 4·93)	0·042
pT category (0 *versus* 1 *versus* 2 *versus* 3 *versus* 4)	2·14 (1·43, 3·21)	< 0·001	1·81 (1·06, 3·08)	0·029
pN category (0 *versus* 1 *versus* 2 *versus* 3)	1·70 (1·34, 2·15)	< 0·001	1·57 (1·14, 2·17)	0·006
Mandard TRG (good *versus* poor)	2·67 (1·22, 5·86)	0·014	4·28 (1·37, 13·34)	0·012
Differentiation (well/moderate *versus* poor)	2·26 (1·27, 4·02)	0·006	2·71 (1·39, 5·29)	0·004
CRM (negative *versus* positive)	2·46 (1·43, 4·22)	0·001		0·171
Lymph node yield (< 15 *versus* ≥ 15)	0·75 (0·43, 1·31)	0·306		

Values in parentheses are 95 per cent confidence intervals. TTO, transthoracic oesophagectomy; THO, transhiatal oesophagectomy; TRG, tumour regression grade; CRM, circumferential resection margin.

**Table 5:** Cox proportional hazards analysis of factors associated with disease‐free survival

	Univariable analysis		Multivariable analysis	
	Hazard ratio	*P*	Hazard ratio	*P*
Age (< 65 *versus* 66–75 *versus* > 75 years)	0·95 (0·59, 1·53)	0·823		
Sex (F *versus* M)	1·08 (0·49, 2·38)	0·847		
Operative approach (TTO *versus* THO)	2·64 (1·36, 5·11)	0·004	3·10 (1·58, 6·12)	0·001
Neutrophil to lymphocyte ratio (< 2·25 *versus* ≥ 2·25)	2·48 (1·08, 5·67)	0·032		0·288
pT category (0 *versus* 1 *versus* 2 *versus* 3 *versus* 4)	1·54 (1·09, 2·17)	0·014	1·72 (1·01, 2·93)	0·047
pN category (0 *versus* 1 *versus* 2 *versus* 3)	1·17 (0·98, 1·40)	0·081		0·091
Mandard TRG (good *versus* poor)	3·68 (1·12, 12·08)	0·032		0·936
Differentiation (well/moderate *versus* poor)	1·82 (0·94, 3·53)	0·078		0·372
CRM (negative *versus* positive)	1·38 (0·69, 2·74)	0·361		
Lymph node yield (< 15 *versus* ≥ 15)	0·71 (0·37, 1·36)	0·302		

Values in parentheses are 95 per cent confidence intervals. TTO, transthoracic oesophagectomy; THO, transhiatal oesophagectomy; TRG, tumour regression grade; CRM, circumferential resection margin.

**Figure 2:**
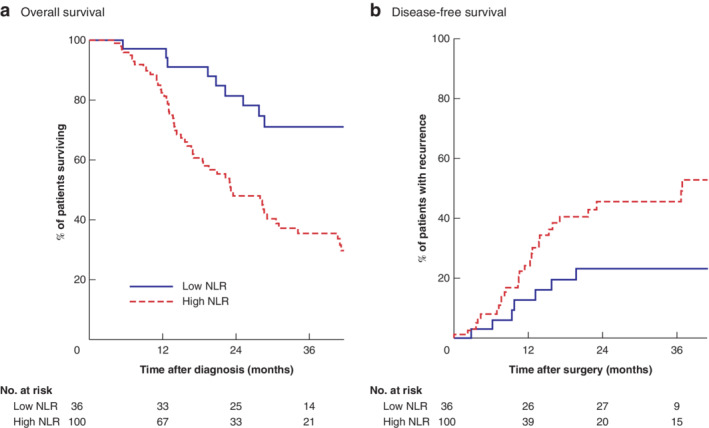
**Kaplan–Meier analysis of overall and disease‐free survival in relation to neutrophil to lymphocyte ratio** **a** Overall and **b** disease‐free survival. NLR, neutrophil to lymphocyte ratio. **a** *P* = 0·001, **b** *P* = 0·027 (log rank test).

## Discussion

The principal finding of this study is that a single biomarker of SIR, a raised NLR, was a significant and independent prognostic indicator of response to NAC before potentially curative oesophagectomy for cancer. Based on a dichotomization threshold of 2·25, 100 of the 136 patients (73·5 per cent) had a raised NLR, and were nearly sixfold more likely to have a poor TRG response to NAC. Median overall survival in patients with a low NLR was, on average, 34 months longer than that in patients with a high NLR. Similarly, 5‐year disease‐free and overall survival rates in patients with a low NLR were around 80 and 50 per cent respectively, approximately double those of patients with a high NLR.

The relationship between the SIR and TRG in oesophageal cancer has been described previously^23^, in a study in which no association between NLR and TRG was identified. Key differences in methodology from that in the present study may account for these different findings. The definition of good TRG response differed. In the earlier study^23^, patients with TRG 1–3 were considered good responders, whereas in the present study TRG 1–2 was considered to represent a good response. The OCCAMS research consortium currently favours the TRG 1–2 as indicative of good response^5^. The statistical methods also differed. The earlier study^23^ looked only at differences in NLR measurements between responders and non‐responders (2·26 *versus* 2·73; *P* = 0·127), but did not examine the predictive value of NLR by ROC curve or logistic regression analysis. Statistical nuances may, of course, be overanalysed, but half of the TRG responders in the earlier study had a NLR below 2·26, which implicitly supports the critical threshold of 2·25 employed in the present study. At the very least, markers of SIR require further evaluation.

The prognostic power of SIR in relation to neoadjuvant therapies has been reported previously, involving rectal^24^, ^25^, ovarian^26^, lung^27^ and breast^28^ cancers. A high mGPS was associated with poor response to NAC in rectal cancer (odds ratio (OR) 0·18, *P* = 0·006), and low NLR was associated with a good pathological response (OR 0·27, *P* = 0·046), although this did not retain independent significance in multivariable analysis^24^. Although other studies have not specifically examined the role of SIR's association with pathological response to NAC, preoperative NLR has been reported to predict overall survival, with high NLR values being associated with poor survival^29^. Exactly why SIR should be associated with poor response to neoadjuvant chemotherapy in OAC is not understood, although *in vivo* and *in vitro* evidence suggests that activation of the JAK/STAT3 (Janus kinase/signal transducers and activators of transcription) pathway by interleukin 6 may play a role in chemoresistance^30^, ^31^.

There are a number of inherent limitations to all studies of this type, which have been reported previously^7^, ^32^, ^33^. Cohort size was modest, and stage‐by‐stage subanalysis was therefore impractical. The patients represented a selected cohort (most had undergone a potentially curative oesophagogastrectomy) and were consequently not representative of all patients diagnosed with oesophageal cancer; indeed, only about one‐quarter of all patients in South Wales with OAC undergo potentially curative surgery^34^. The strengths of the study, nevertheless, included robust follow‐up data with no patient lost to follow‐up, a reasonable duration of follow‐up, and accurate causes and dates of death. A National Health Service laboratory using standardized techniques performed the serum analyses and histopathological examinations, so reproducing these results should be easy. Patients were recruited from a consecutive series diagnosed with OAC, from a single UK geographical region, all treated by the same group of specialists, using a standardized staging algorithm and operative techniques, with internationally recognized and published key performance indicators^10^.

Despite improvements in staging and surgical technique, approximately half of the patients who undergo potentially curative oesophagectomy for cancer will suffer disease recurrence^13^, ^22^. Determination of the NLR, derived and calculated from absolute counts of serum lymphocytes and neutrophils, is performed routinely during preoperative blood profile work, and is readily available. The findings suggest that SIR offers a novel therapeutic target for patients susceptible to NAC resistance and cancer recurrence. Incorporation of the NLR into management pathways is presently limited by inconsistent dichotomization thresholds. Adequately powered studies comparing critical dichotomization or categorization thresholds are needed. Given the association between SIR and relative chemoresistance, the identification of the group with high NLR would suggest that these patients might benefit from alternatives to NAC at the outset.

## Collaborators

Members of the South‐East Wales Oesophagogastric Cancer Collaborative: G. Blackshaw, G. Clark, X. Escofet, A. Foliaki, T. Havard, M. Henwood, J. Witherspoon, W. G. Lewis.

## Disclosure

The authors declare no conflict of interest.
